# Use of healthcare reimbursement data to monitor bacterial sexually transmitted infection testing in France, 2006 to 2020

**DOI:** 10.2807/1560-7917.ES.2022.27.39.2100618

**Published:** 2022-09-29

**Authors:** Delphine Viriot, Etienne Lucas, Bertille de Barbeyrac, Cécile Bébéar, Sébastien Fouéré, Nicolas Dupin, Antoine Bertolotti, Béatrice Berçot, Charles Cazanave, Gilles Delmas, Josiane Pillonel, Florence Lot, Ndeindo Ndeikoundam Ngangro

**Affiliations:** 1 Santé publique France (the French National Public Health Agency), Saint-Maurice, France; 2 French National Reference Centre for bacterial STI (Chlamydia, Mycoplasma), Bordeaux University Hospital, Bordeaux, France; 3 APHP, Saint-Louis University Hospital, Paris, France; 4 French National Reference Centre for bacterial STI (Syphilis), APHP, Cochin University Hospital, Paris, France; 5 Inserm-CIC1410, La Reunion University Hospital, Saint Pierre, Reunion Island, France; 6 French National Reference Centre for bacterial STI (Gonorrhea), APHP, Saint-Louis University Hospital, Paris, France; 7 Bordeaux University Hospital, Bordeaux, France

**Keywords:** STI, chlamydia infection, gonorrhoea, syphilis, testing, COVID-19 impact

## Abstract

**Background:**

Diagnoses of bacterial sexually transmitted infections (STIs) have increased in France since the 2000s. The main strategy to control STI transmission is recommending/facilitating access to condom use, testing, and antibiotic treatments.

**Aim:**

This study analyses the evolution of STI testing in the private sector in France from 2006 to 2020.

**Methods:**

National health insurance reimbursement data were used to determine numbers and rates of individuals aged ≥ 15 years tested for diagnoses of chlamydia, gonorrhoea and syphilis in the private sector in France and to describe their evolution from 2006 to 2020.

**Results:**

Upward tendencies in testing were observed from 2006 to 2019 for all three STIs. The highest testing rates were identified in people aged 25‒29-years old. The observed testing-increase from 2017 to 2019 was twice as high in young people (< 25 years old) as in older people. In 2019, chlamydia, gonorrhoea and syphilis testing rates were respectively 45.4 (+ 21% since 2017), 41.3 (+ 60%), and 47.2 (+ 22%) per 1,000 inhabitants. For all STIs combined, the number of tested individuals decreased by 37% between March and April 2020 during the first COVID-19 epidemic wave and lockdown in France.

**Conclusion:**

Improvements found in STI testing rates may have resulted from better awareness, especially among young people and health professionals, of the importance of testing, following prevention campaigns. Nevertheless, testing levels remain insufficient considering increasing diagnoses. In 2020, the COVID-19 pandemic had a considerable impact on STI testing. Partner notification and offering diverse testing opportunities including self-sampling are essential to control STI epidemics particularly in exposed populations.

## Introduction

It has been estimated that nearly 200,000 chlamydia and 50,000 gonorrhoea diagnoses are made annually in France [1]. Considering these high numbers, the main strategies to reduce the transmission risk of bacterial sexually transmitted infections (STIs) consist in encouraging partner notification, as well as recommending and facilitating access to condom use and testing. Screening is also important to prevent complications as it enables early diagnosis and treatment [2].

From 2003 to 2018, systematic screening for *Chlamydia trachomatis* was recommended in France for women under 25 years old and men under 30 years old, in medical facilities specialised in the diagnostic and therapeutic management of this STI, such as anonymous and free screening centres [3]. Subsequently, other diagnostic centres were targeted by the recommendation including in the private sector [1,3,4]. Since September 2018, chlamydia screening has been recommended for all sexually active women aged between 15 and 25 years as well as for women and men with risk factors, regardless of age, including people with multiple partners or new partners, sex workers and men who have sex with men (MSM) [4].

Opportunistic testing for syphilis and gonorrhoea targets populations at risk of STIs through engaging in unprotected sex, such as MSM, people with multiple sexual partners, people with an antecedent of STI and people whose sexual partner is diagnosed with an STI. Moreover, screening for syphilis is mandatory during the first trimester of pregnancy [5,6].

While these recommendations are usually followed, in 2020 the coronavirus disease (COVID-19) pandemic had a considerable impact on the use of the healthcare system and on the access to testing in particular.

To contribute to the evaluation of the French STI testing strategy, this article uses national healthcare reimbursement data to analyse the evolution of testing for chlamydia, gonorrhoea, and syphilis in the private sector from 2006 to 2020.

## Methods

### Study population and data source

Individuals aged 15 years and over who were reimbursed for STI testing during the study period from 1 January 2006 to 31 December 2020 were included. The French national health database (*Système national des données de santé*, SNDS) regularly collects data relating to the reimbursement of STI tests in the population (around 63 million beneficiaries or 95% of the total population). In this retrospective study, data provide information on testing in the private sector but exclude testing performed in public hospitals and free testing centres in STI clinics [7].

### Test selection

For chlamydia, several tests were used during the study period. Regarding the gene amplification tests for *C. trachomatis*, nucleic acid amplification tests (NAAT) were selected in the SNDS database by searching the reference code 5257 from the French Nomenclature of Biological Acts (NABM). After 2018, the nomenclature was modified; *C. trachomatis* and/or *Neisseria gonorrhoeae* were screened together using NAAT with the code 5301 [8]. Moreover, the detection of *C. trachomatis* and/or *N. gonorrhoeae* by gene amplification has been reimbursed since September 2019 for two (code 5302) or three sampling sites (code 5303) [8], so these codes were also taken into account. Additional tests relating to microbiological examinations (codes 5202 to 5205), immunoassays (code 5254), culture tests (code 5255), and serological tests (code 1307) were included as well.

For gonorrhoea, only culture of *N. gonorrhoeae* (codes 5202 for female samples and 5203 for male samples) was reimbursed until June 2018. Thereafter, NAAT testing was included in the NABM (code 5301). These three codes were thus selected along with codes 5302 (two sampling sites) and 5303 (three sampling sites) [8].

For syphilis, a combination of treponemal (TT) and non-treponemal tests (NTT) was recommended to detect infections until 2018 [5]. Thus, the combined reimbursement codes corresponding to prescriptions for syphilis screening and treatment guidelines [9-11] were selected. Serological tests with two reactions, at least one from each of the following groups, including group 1 (NTT venereal disease research laboratory (VDRL)) and group 2 (TT: *Treponema pallidum* haemagglutination (TPHA), antibody enzyme immunoassay (EIA), fluorescent treponemal antibody test absorption (FTA abs)) were under detection code 1326. In case of a positive or dissociated reaction, a titration must be performed on each group, i.e. for the two titrations, and this was under code 1327. Moreover, supplementary codes for confirmation of the infection were also taken into account: immunoglobulin (Ig)M immunoassays code 1330, tests to detect T. pallidum directly (darkfield examination or other test code 0246 or 5291).

Following changes to the NABM in 2018, the systematic combination of TT and NTT was replaced by a single TT, with total Ig confirmed by quantitative NTT in the case of positivity [8]. Codes 1256, 1257, and 1258 were respectively used for total Ig test, NTT with titration, and a complementary NTT in the case of suspected seroconversion [8,9].

### Analyses

The testing rate for each pathogen was defined as the number of people 15 years and older with at least one reimbursement for STI testing per year divided by the number of people 15 years and older in the French population using census data (from the National Institute of Statistics and Economic Studies; INSEE). These rates were calculated by sex (male/female) and age at the national and regional levels. Inter-regional analysis was performed by considering three entities: the French overseas departments (FOD), the Paris area or Île-de-France (IDF), and the remaining regions of metropolitan France. Regional rates were estimated by direct standardisation related to age and sex (to allow for inter-regional comparisons of rates taking into account population structure by age and sex). Epidemiological trends were described from 2006 to 2020, with a particular focus on the impact of COVID-19 in 2020.

The characteristics of testers were described in 2019, which was the most recent year before the COVID-19 pandemic. Trends were compared using a chi-squared trend test. SAS Enterprise guide (v. 4.3) and STATA (v.15) were used for statistical analysis.

## Results

### Chlamydia

**Figure 1:**
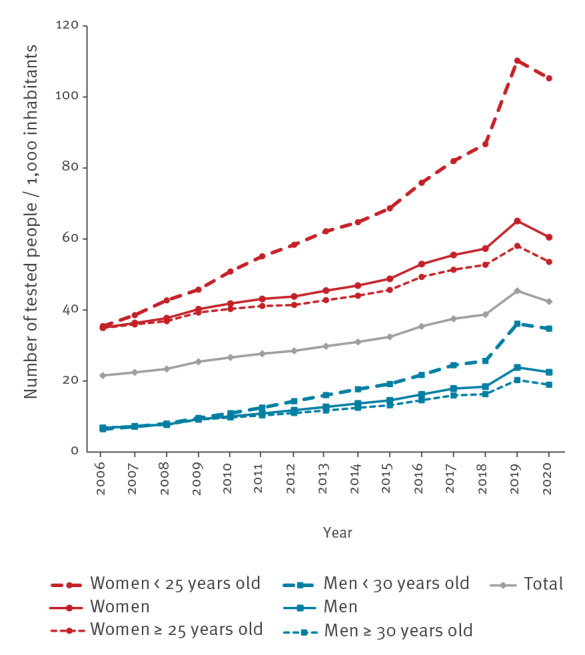
Testing rates for chlamydia per 1,000 inhabitants by year, sex, and age group, National Health Data System, France, 2006–2020 (n = 25,146,456 individuals tested)

From 2006 to 2019, an average of 1.7 million tests (range: 1.1–2.5) were performed each year. The testing rate climbed steadily between 2006 and 2015 (around + 5% annually) and then more markedly over the 2016 year (+ 9%) (Figure 1). Between 2017 and 2019, a 21% increase of the testing rate was observed: this rise was stronger in men (+ 33%) than in women (+ 17%) (p < 0.001). However, by age group, the increase was more than double for young people under 25 years of age (+ 38%) compared with those aged 25 years and older (+ 17%) regardless of sex (p < 0.001). The increase over this period was almost three times more for women under 25 years (+ 35%) than in those aged 25 years and older (+ 13%) (p < 0.001). Similarly, the increase was more pronounced for men under 30 years (+ 48%) compared with those aged 30 years and older (+ 27%) (p < 0.001). Comparing the annual rate of 2019 to that of 2020, the testing rate decreased by 7% (− 6% for men vs − 7% for women) in 2020. The decrease was less marked in women under 25 years (− 5%) as well as men under 30 years (− 4%). At the regional level, overall upward tendencies between 2017 and 2019 were noted in FOD, IDF, and other metropolitan French regions, with increases in testing rates of 14%, 19%, and 22%, respectively. From 2019 to2020, testing decreased in IDF (− 6%), FOD (− 9%), and other regions (− 7%).

Rates per 1,000 inhabitants in the Figure represent the number of people tested (according to their sex, or sex and age group) among the number of inhabitants of the same respective sex, or sex and age group. All rates shown are for people aged 15 years and older.

In 2019, 2.5 million people were tested, the majority of whom were women (75%; 1,868,815/2,493,962). The national testing rate was 45.4 per 1,000 inhabitants aged 15 years and older (Figure 1). This rate was three times higher among women (65.1 per 1,000) than among men (23.8 per 1,000) (p < 0.001). The highest testing rates were observed among women and men aged 25 to 29 years (191.9 and 57.5 per 1,000, respectively) and among men aged 30 to 34 years (57.0). The rates were 110.3 per 1,000 among women under 25 years old and 36.1 per 1,000 among men under 30 years old. The testing rate for people aged under 25 years (67.4 per 1,000) was half that of 25–29-year-olds (125.5) (p < 0.001) regardless of sex.

**Figure 2:**
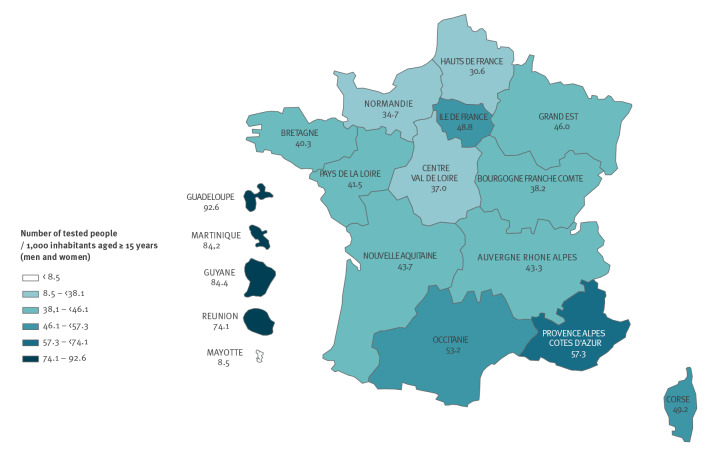
Standardised testing rates for chlamydia by region, National Health Data System, France, 2019 (n = 2,493,962 individuals tested)

In 2019, in metropolitan France, testing rate levels were the highest in five regions: Provence-Alpes-Côte d’Azur (57.3 per 1,000 inhabitants), Occitanie (53.2), Corsica (49.2), IDF (48.8), and Grand Est (46.0). In FOD except for Mayotte, the testing rates were approximately twice as high as in metropolitan France (43.4), reaching 92.6 per 1,000 inhabitants in Guadeloupe (p < 0.001) (Figure 2).

SNDS: Système national des données de santé (French national health database).

Source: données SNDS 2019, Exploitation Santé Publique France; Mars 2022.

### Gonorrhoea

**Figure 3:**
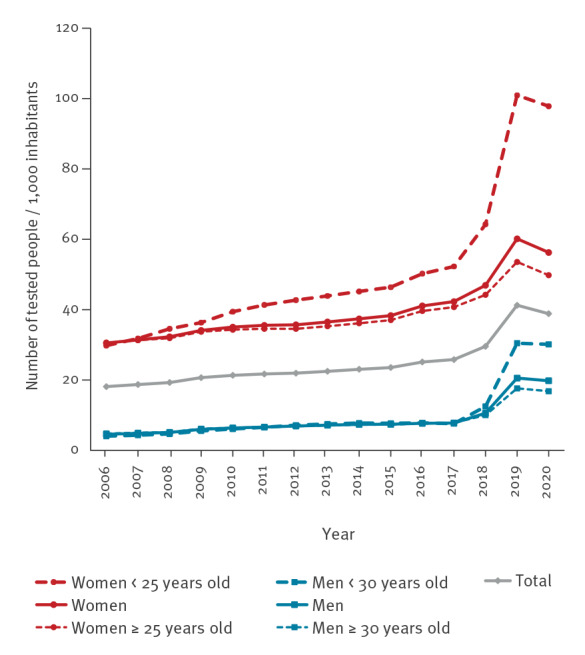
Testing rates for gonorrhoea per 1,000 inhabitants by year, sex, and age group. National Health Data System, France, 2006–2020 (n = 19,948,443 individuals tested)

From 2006 to 2019, an average of 1.3 million tests (range: 0.9–2.3) were performed each year. The testing rate increased steadily between 2006 and 2015 (around + 3% annually) and then more markedly over the 2016 year (+ 7%). Between 2017 and 2019 there was a sharp rise (+ 60%) compared with 2017: the increase was four times higher in men (+ 164%) than in women (+ 42%) (p < 0.001) (Figure 3). In this same period, the rise was more than double for young people under 25 years of age (+ 118%) compared with those aged 25 years and older (+ 49%) regardless of sex (p < 0.001). For women under 25 years, the increase was almost three times (+ 93%) that of those aged 25 years and older (+ 31%) (p < 0.001). Similarly, the increase was more pronounced for men under 30 years (+ 300%) compared with those aged 30 years and older (+ 125%) (p < 0.001). From 2019 to 2020, the testing rate decreased by 5.8% (− 3.7% for men vs − 6.5% for women). The decreases were less marked in women under 25 years (− 3%) and men under 30 years (− 1%). At the regional level, upward tendencies were observed in IDF (+ 58%), FOD (+ 54%), and other regions (+ 60%) between 2017 and 2019. From 2019 to 2020, a decrease was observed in IDF (− 5%), FOD (− 8%), and other regions (− 5%).

Rates per 1,000 inhabitants in the Figure represent the number of people tested (according to their sex, or sex and age group) among the number of inhabitants of the same respective sex, or sex and age group. All rates shown are for people aged 15 years and older.

In 2019, 2.3 million people were tested, with a national testing rate of 41.3 per 1,000 inhabitants (Figure 3). The majority of tested individuals were women (76%; 1,727,594/2,266,344), whose testing rate was three times higher than that of men (60.2 vs 20.5 per 1,000; p < 0.001). The highest rates were observed among women aged 25 to 29 years (178.0 per 1,000) and men aged 30 to 34 years (50.2 per 1,000). The rates among women under 25 years old were 101.0 per 1,000 and among men under 30 years old 30.5 per 1,000. The testing rate among people under 25 years old (60.5 per 1,000) was much lower than that among 25–29-year-olds (114.6) regardless of sex (p < 0.001).

**Figure 4:**
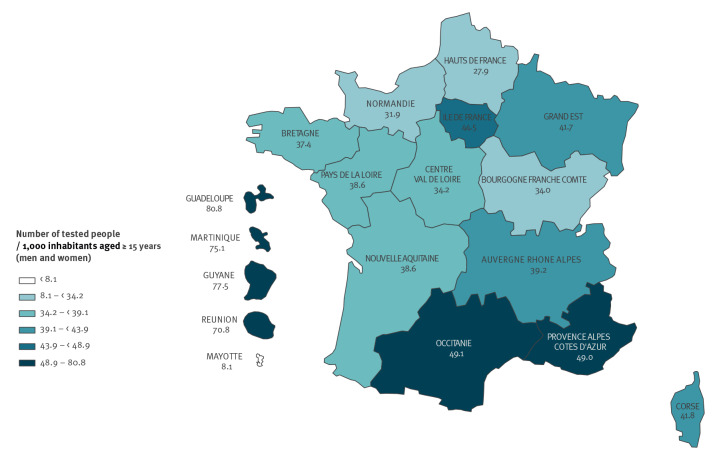
Standardised testing rates for gonorrhoea by region, National Health Data System, France, 2019 (n = 2,266,344 individuals tested)

In 2019 in metropolitan France, the highest testing rates were observed in Occitanie (49.1 per 1,000 inhabitants), Provence-Alpes-Côte d’Azur (49.0), IDF (44.5), Corsica (41.8), and Grand Est (41.7) in 2019. Testing rates in FOD except for Mayotte exceeded the national rate and were double those recorded in metropolitan France (39.1), reaching 80.8 per 1,000 inhabitants in Guadeloupe (p < 0.001) (Figure 4).

SNDS: Système national des données de santé (French national health database).

Source: données SNDS 2019, Exploitation Santé Publique France; Mars 2022.

### Syphilis

From 2006 to 2019, an average of 1.6 million tests (range: 1.0–2.6) were performed each year. The testing rate increased steadily between 2006 and 2015 (around + 6% annually) and then more markedly over the 2016 year (+ 10%) and also over 2017 (+8%). Between 2017 and 2019, the testing rate rose by + 22% overall (+ 31% among men vs + 18% among women; p < 0.001). In this same period, the increase was more than double for young people under 25 years of age (+ 39%) compared with those aged 25 years and older (+ 18%) regardless of sex (p < 0.001). The testing rate among women under 25 years old had an increase by 35%, more than double that observed in those aged 25 years and older (+ 14%) (p < 0.001). The testing rate among men under 30 years increased by 43% compared with only 26% for those aged 30 years and older (p < 0.001). From 2019 to 2020, the testing rate decreased by 6% (− 10% for men vs − 5% for women). At the regional level, between 2017 and 2019, the observed testing rate increased by + 19% in IDF, + 21% in FOD, and + 23% in other regions. From 2019 to 2020, a decrease was observed in IDF (− 5%), FOD (− 9%), and other regions (− 6%).

**Figure 5:**
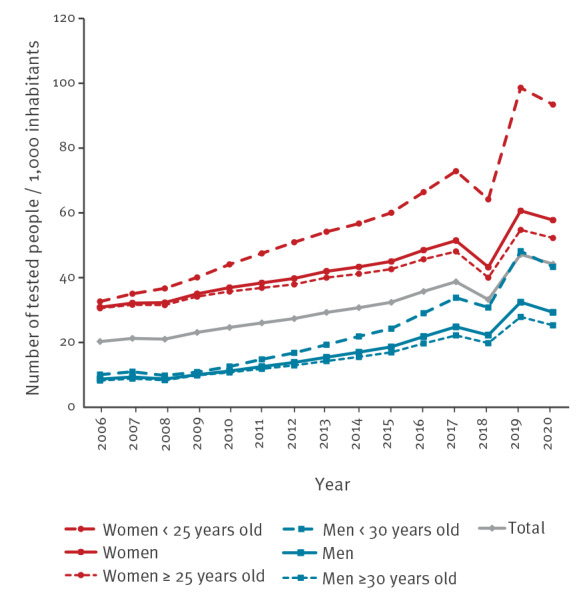
Testing rates for syphilis per 1,000 inhabitants by year, sex, and age group. National Health Data System, France, 2006–2020 (n = 24,461,232 individuals tested)

In 2019, 2.6 million people were tested, the majority of whom were women (67%; 1,741,886/2,592,522) (Figure 5). The national testing rate was 47.2 per 1,000 inhabitants. The rates by sex were, among women, 60.7 per 1,000 and, among men, 32.4 per 1,000. The highest testing rates by sex were observed in women aged 25 to 29 years (203.8 per 1,000) and men aged 25 to 29 years (71.9). The testing rate for people under 25 years old (67.3 per 1,000) was much lower than that of 25–29-year-olds (138.7) (p < 0.001).

The decrease observed in 2018 does not correspond to a real decrease in the number of screenings but to a difficulty in reporting data at the level of health insurance.

Rates per 1,000 inhabitants in the Figure represent the number of people tested (according to their sex, or sex and age group) among the number of inhabitants of the same respective sex, or sex and age group. All rates shown are for people aged 15 years and older.

**Figure 6:**
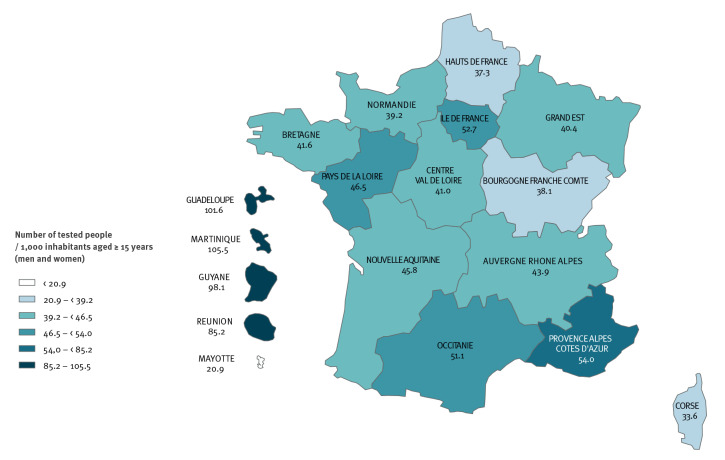
Standardised testing rates for syphilis by region, National Health Data System, France, 2019 (n = 2,592,522 individuals tested)

In 2019 in metropolitan France, the testing rates were the highest in Provence-Alpes-Côte-D’Azur (54.0 per 1,000 inhabitants), IDF (52.7), and Occitanie (51.1). Testing rates in FOD except for Mayotte were twice as high as those recorded in metropolitan France (54.3), reaching 105.5 per 1,000 inhabitants in Martinique (p < 0.001) (Figure 6).

SNDS: Système national des données de santé (French national health database).

Source: données SNDS 2019, Exploitation Santé Publique France; Mars 2022.

### Impact of coronavirus disease on bacterial sexually transmitted infection testing in 2020

In France in 2020, the first wave of the COVID-19 pandemic occurred from March to May (with a peak in April) followed by a second wave from October to November (with a peak in late October). During the first wave of COVID-19, the total number of screenings in April 2020 decreased by 37% compared to that of March 2020 (− 40% for chlamydia, − 39% for gonorrhoea, and − 32% for syphilis), whereas the number of screenings had remained more or less stable in the same period in 2019 (− 6% for these three STIs respectively between March and April 2019). The decreases were more marked in men (− 49%) than in women (− 32%), especially for syphilis (− 49% vs − 24%) but also for chlamydia (− 49% vs − 37%) and gonorrhoea (− 49% vs − 36%) (p < 0.001). A rise in testing then followed from April to June 2020 (+ 78%). However, from June to August 2020, the screening volumes seemed to decrease once again (− 15%). From September to December 2020, the observed decline continued (− 46% for chlamydia, − 46% for gonorrhoea, and − 43% for syphilis) but in a similar way to the previous year (− 46% for chlamydia, − 45% for gonorrhoea, and − 42% for syphilis from September to December 2019). The lowest rates were recorded in December but at a level similar to those observed in the previous year in the same period. Overall in 2020, the total number of STI screenings was 6% less compared with 2019.

## Discussion

With 2.5 million individuals tested for chlamydia, 2.3 million for gonorrhoea, and 2.6 million for syphilis in 2019, this study highlights the importance of private laboratories for STI testing in France.

Our values for syphilis and gonorrhoea concur with those obtained in 2016 by a French national survey on STI testing in the private sector, which found 2.5 and 1.8 million people respectively tested for these two diseases. For chlamydia, however, the number reported here is lower than the 3.8 million tested people quantified by the survey (data not shown).

From 2006 to 2019, we observed that testing rates for all three STIs showed an upward tendency. The increases in chlamydia (+ 21%) and syphilis (+ 22%) testing between 2017 and 2019 are consistent with increases in HIV testing in France (+ 16% between 2017 and 2019), with 6.2 million HIV serological tests performed in 2019 (76% in private laboratories) [12]. During the same period, the rise in gonorrhoea testing (+ 60%) was much higher and further evolution in the coming years will be the subject of particular attention. This finding may partly reflect improvements in access to testing and better awareness of the population on the importance of testing following prevention campaigns. However, it may also result from more frequent risky sexual behaviours and inadequate reduction of STI transmission, with testing levels still remaining insufficient. The overall upward tendency in STI testing follows increases of cases in the context of an STI epidemic in France, which has been observed through surveillance since the 2000s [13]. More recently, a sharp increase in the rate of chlamydia diagnoses was also confirmed by another study in the private sector from 2017 to 2019 (+ 30%) [14].

For the three STIs concerned in this report, the majority of tested people were women**,** in line with previous data [15]. From 2017 to 2019, while testing rates tended to rise regardless of sex, increases in testing were generally higher in men. This is possibly due to more testing among men particularly at risk for STIs, who are more specifically targeted by recommendations since 2018. The increases in younger women (under 25 years old) and younger men (under 30 years old) were greater than in older individuals. These testing tendencies for young people are consistent with the national recommendation for chlamydia screening in women under 25 years and men under 30 years [3].

From a geographical perspective, the data reveal strong regional disparities. In FOD except for Mayotte, the testing rate is almost double that of metropolitan France. In metropolitan France, the testing rate is higher in the southern regions and IDF. The highest diagnosis rates are also observed in FOD, IDF, and southern France [1], which could be linked to both the high testing rate and the high STI prevalence in these areas. The reasons for lower testing rates in some areas, such as northern France and Mayotte are important to understand as they may reflect differences in terms of availability of healthcare and access to it.

This analysis revealed a steady increase in testing for chlamydia from 2006 to 2015 followed by a more marked increase in 2016. This trend observed in 2016 could partly be due to a prevention campaign implemented in response to the resurgence of STIs, as well as to the testing recommendations of the French Dermatology Society whereby the partners of positive cases should benefit from chlamydia screening [16,17]. Between 2017 and 2019, the increase in chlamydia testing in men under 30 years was almost double that observed in men aged 30 or older. Similarly, the increase over this period was approximately three times higher for women under 25 years compared with older ones. This finding points to improved testing of these younger populations, even though the private sector was not targeted by testing recommendations before 2018 [1,3,4]. Nevertheless, in 2019 the rates observed in women under 25 years and men under 30 years were about two times lower than those observed in women aged 25 to 29 years and men aged 30 to 34 years, respectively. Testing thus remains insufficient in young people, especially given that this population is particularly affected by bacterial STIs [18].

Comparisons with other countries are complex, since limited testing data have been published, with substantial variations being observed between countries in terms of the testing and notification of cases for Chlamydia [18]. Our data may be compared with testing rates of non-specialised facilities in England, although online testing is also available in England [15]. In 2019, testing in France (45.4 per 1,000 inhabitants, + 21% compared with 2017) is comparable to testing performed in non-specialised structures in England (41.6 per 1,000, + 15% in England from 2017 to 2019) [19]. Nevertheless, in 2018 in Denmark, a higher rate was observed (88.9 per 1,000), with lower testing rates among men aged 15 years and over (48.5 per 1,000) than among women of the same age category (129.3 per 1,000) [20]. A high testing rate was also observed in Sweden (607,062 tests in 2019, or 71 per 1,000 inhabitants) [21].

In France, a steady increase in gonorrhoea testing was observed until 2017, although culture was the only reimbursed test. Subsequently, a substantial increase occurred in 2018, which can partly be attributed to the reimbursement of NAAT since June 2018. The increase in testing was greater for women under 25 years and for men under 30 years compared with older individuals. Nevertheless, in 2019, the rates observed in women under 25 years and men under 30 years were lower than those observed in women aged 25 to 29 years and men aged 30 to 34 years, respectively, which highlights the need to provide testing solutions that are better adapted to younger populations [1].

In England, the total testing rate for gonorrhoea was equal to 45.0 per 1,000 inhabitants in 2019 with an upward trend being observed since 2017 (+ 20% vs + 60% in France) [19]. This total rate (in specialised and non-specialised facilities) in England is almost equivalent to the testing rate in the private sector alone in 2019 in France (41.3). Considering that this sector represents 84% of total gonorrhoea testing in the country (data not shown), it can be deduced that the overall testing rate appears higher in France than in England. An even higher rate was observed in Sweden (544,795 tests in 2019, or 64 per 1,000 inhabitants) [21].

In France, a steady increase in testing for syphilis was observed from 2006 to 2017, with a subsequent sharper apparent rise between 2017 and 2019. It should be noted that a decrease observed in the meantime, in 2018, was due to a technical issue in the reporting of data at the national health insurance level. In 2019, the increase in the testing rate was high for people under 25 years. However, the testing rate for people under 25 was much lower than that of 25–29-year-olds.

The high proportion of women tested for syphilis in 2019 (i.e. over 65%) is due to the mandatory antenatal screening for this STI. As a comparison, 1.5 million syphilis tests, including ca 750,000 tests for women (excluding antenatal screening), were performed in England in 2019 (specialised and non-specialised facilities, + 18% compared with 2017) [19]. With nearly 700,000 antenatal tests carried out in a single year [22], the total testing rate in England may be estimated at 48.1 per 1,000 inhabitants, which is similar to the French data given that the private sector (47.2 per 1,000 inhabitants) represents 94% of the total syphilis testing in France (data not shown).

During the first wave of the COVID-19 pandemic from March to May 2020, a sharp decrease in screening for chlamydia, gonorrhoea and syphilis was observed in France, particularly in April (peak during the first lockdown). This was the first time that such a decrease had been observed at this time of year. It was less marked in women for syphilis testing compared with chlamydia and gonorrhoea testing, probably due to the mandatory antenatal screening for syphilis.

The decrease in screening observed from March to May 2020, was not subsequently compensated for according to consolidated data up to December 2020. The number of HIV serological tests performed in French private laboratories also decreased by 37% from March to May 2020 (− 44% in men and − 34% in women) compared with the same period in 2019 [12]. Despite the substantial decrease during the first lockdown, the total number of STI screenings in 2020 was only 6% less than in 2019, whereas HIV tests decreased by almost 10% from 2019 to 2020. However, as with HIV, this trend may have given rise to concerns about delays in screening and appropriate treatment, with an increased risk of circulation of these STIs.

In a COVID-19 survey to assess impact of the pandemic on testing for HIV, viral hepatitis, and STIs in the World Health Organization (WHO) European Region, 95% of the 98 survey respondents from 34 countries (23 European Union/European Economic Area (EU/EEA) and 11 non-EU/EEA) reported a decrease in testing during the March–May period and 58% during the June–August period in 2020 compared with the previous year. Testing decreased by more than 50% in the majority of the countries (64%) during the March–May period and in 20% of countries during the June–August period [23]. Similar decreases were observed in England for these STIs during the lockdown period (from − 52.1% to − 58.7% from March to April) [24]. A decrease of 40% was observed in the United States (− 59% in women vs − 63% in men) when comparing the pandemic period (from March to June 2020) and the preceding baseline period for chlamydia and gonorrhoea [25]. The overall decrease (− 6%) in France in 2020 is consistent with the decline from 2019 to 2020 observed in Sweden in diagnostic testing (− 10.5% for chlamydia, − 9.4% for gonorrhoea) [21]. Moreover, there are indications that, during the COVID-19 outbreak in 2020, considerably less STIs were diagnosed in several countries [26-28], but only few testing-result data have been published, such as the decreases in diagnosed STIs reported in Greece [26] and Spain [27]. It is also interesting to note that an upward trend in diagnoses was observed in a few countries like Italy and Finland [28], probably linked to the different restrictions or communications relating to COVID-19.

For the period from September to December 2020, testing trends in France were similar to those of the previous years. The second lockdown implemented in October for the second wave of COVID-19 had a lesser impact on STI testing. This could be explained by the implementation of less restrictive measures compared with the previous lockdown.

This study has some limitations. The first limitation is that reimbursement data from the SNDS database exclude testing conducted in public hospitals and free testing centres in STI clinics. Thus ca 10% of the total number of laboratories in France are not included in the current analysis, corresponding to 9% of the total number of tests for chlamydia, 16% for gonorrhoea, and 6% for syphilis (data not shown). Accordingly, the indicators reported in our study do not account for the total screening conducted in the country. Second, the reimbursement of tests by gene amplification in specimens from two (code 5302) or three (code 5303) anatomical sites, only occurred since September 2019 for chlamydia and gonorrhoea. As a result, the number of tests performed before this date is probably underestimated. Third, the data from SNDS also do not provide information about sexual behaviour. Consequently, it is not possible to identify the contribution of specific at-risk populations (MSM, people with multiple sexual partners, etc.) targeted by national recommendations for STI screening. Moreover, it would be interesting to have information relating to pre-exposure prophylaxis (PrEP), which was introduced in France in 2016 to reduce the risk of contracting HIV. Further analyses relying on algorithm identifying PrEP users might help to better understand the impact of PrEP on STI testing. The use of this preventive treatment could have an impact on testing strategies by requiring regular medical follow-up. Several meta-analyses show that the use of PrEP was associated with an increase in bacterial STIs in MSM [16,29]. Fourth, the SNDS database neither contains test result information nor clinical data. Consequently, it is necessary to build algorithms to identify cases using test and treatment reimbursement data.

## Conclusion

Our data show an overall increase in testing for chlamydia, gonorrhoea, and syphilis in the French private sector from 2006 to 2019, with a more substantial increase in the testing rate in people under 25 years since 2016. This may result from better awareness among the general population and health professionals following prevention campaigns.

In France, the increase in the number of diagnoses of bacterial STIs may partly result from the increase in testing up to 2019, although the upward trend in bacterial STIs also points to the need to further improve testing practices to control STI epidemics. The current testing offer was expanded in 2018 with the implementation of new screening recommendations for chlamydia that target both general practitioners and specialised facilities. In the current context of the uncontrolled spread of bacterial STIs, condoms are the only primary prevention method. In the event of unprotected sex, testing is essential to initiate effective treatment and break transmission chains.

In 2020, the COVID-19 pandemic had a considerable impact on STI testing. New measures such as remote consultations, self-sampling, and appointment-only testing were implemented in some European countries to reduce the impact of COVID-19 on STI testing [21]. In the context of the SARS-CoV-2 pandemic, it is therefore necessary to adapt the healthcare system and encourage the development of these alternative strategies.
