# Author's correction for Euro Surveill. 2022;27(39)

**DOI:** 10.2807/1560-7917.ES.2022.27.42.221020c

**Published:** 2022-10-20

**Authors:** Eurosurveillance editorial team

**Affiliations:** 1 European Centre for Disease Prevention and Control (ECDC), Stockholm, Sweden

In the article ‘Use of healthcare reimbursement data to monitor bacterial sexually transmitted infection testing in France, 2006 to 2020’ by Viriot et al., published on 29 September 2022, the number ’43.5’ was mistyped as ’54.3’ in one sentence. This was corrected on 18 October 2022 at the request of the authors.

